# Epithelioid hemangioendothelioma of the retroperitoneal giant type treated with Toripalimab: A case report

**DOI:** 10.3389/fimmu.2023.1116944

**Published:** 2023-03-17

**Authors:** Yuqing Bu, Lili Peng, Miaomiao Liu, Liya He, Fayan Wang, Bingjie Li, Xueliang Niu, Hongzhen Zhang

**Affiliations:** ^1^ Department of Oncology, Hebei General Hospital, Shijiazhuang, Hebei, China; ^2^ Department of Medicine, Hebei North University, Zhangjiakou, Hebei, China; ^3^ Department of Medical Affairs, Shanghai Junshi Biosciences Co., Ltd., Shanghai, China

**Keywords:** epithelioid hemangioendothelioma, retroperitoneal tumor, immunotherapy, toripalimab, PD-1 inhibitor

## Abstract

Epithelioid hemangioendotheliomas (EHEs), low-grade malignant tumors of vascular endothelial cell origin, are characterized by vascular endothelial proliferation. In 2002, the World Health Organization classified EHEs as locally aggressive tumors with the potential to metastasize. Currently, the diagnosis of EHE is based on pathology, histological and immunohistochemical examinations. There are no standard treatment guidelines. We here report a 69-year-old man who presented with left-sided chest and abdominal pain for more than 2 months. Enhanced computed tomography of the thorax and abdomen in another hospital suggested a mass in the left adrenal region that was considered malignant. Positron emission tomography- computed tomography in our hospital suggested a large multi-loculated, hypermetabolic, cystic mass in the left adrenal region that was considered malignant. Accordingly, a puncture biopsy of the mass was performed and the diagnosis of EHE confirmed by pathological examination, including immunohistochemical staining. This patient was treated with the programmed death 1 (PD-1) immune checkpoint inhibitor toripalimab with long-term success. The best response was stable disease (SD) with a progression-free survival (PFS) of more than 13 months. The patient is still alive now. Because the sample size of previous studies was small, further studies are needed to determine the safety and efficacy of toripalimab in the treatment of EHE.

## Introduction

EHEs usually occur in middle-aged patients, with the median age of 36 years (range approximately 7 to 83 years) (1). These lesions can occur in the liver, lungs, bones, head and neck, breast, mediastinum, and other sites. They rarely occur in the abdominal cavity. It has been reported that patients with EHE can survive up to 24 months; however, some patients survive for only 6 months (2). Because of its insensitivity to various treatments and rapid growing at the late stage, EHE is prone to develop into highly malignant tumors. Therefore, in 2002 the WHO classified EHEs as locally aggressive tumors with the potential to metastasize (3). Because of their unique characteristics, it is difficult to plan optimal treatment (4, 5). We here report the diagnosis and treatment of a retroperitoneal epithelioid hemangioendothelioma with toripalimab and review published reports to provide new clinical insights.

## Case presentation

**Figure 1:**
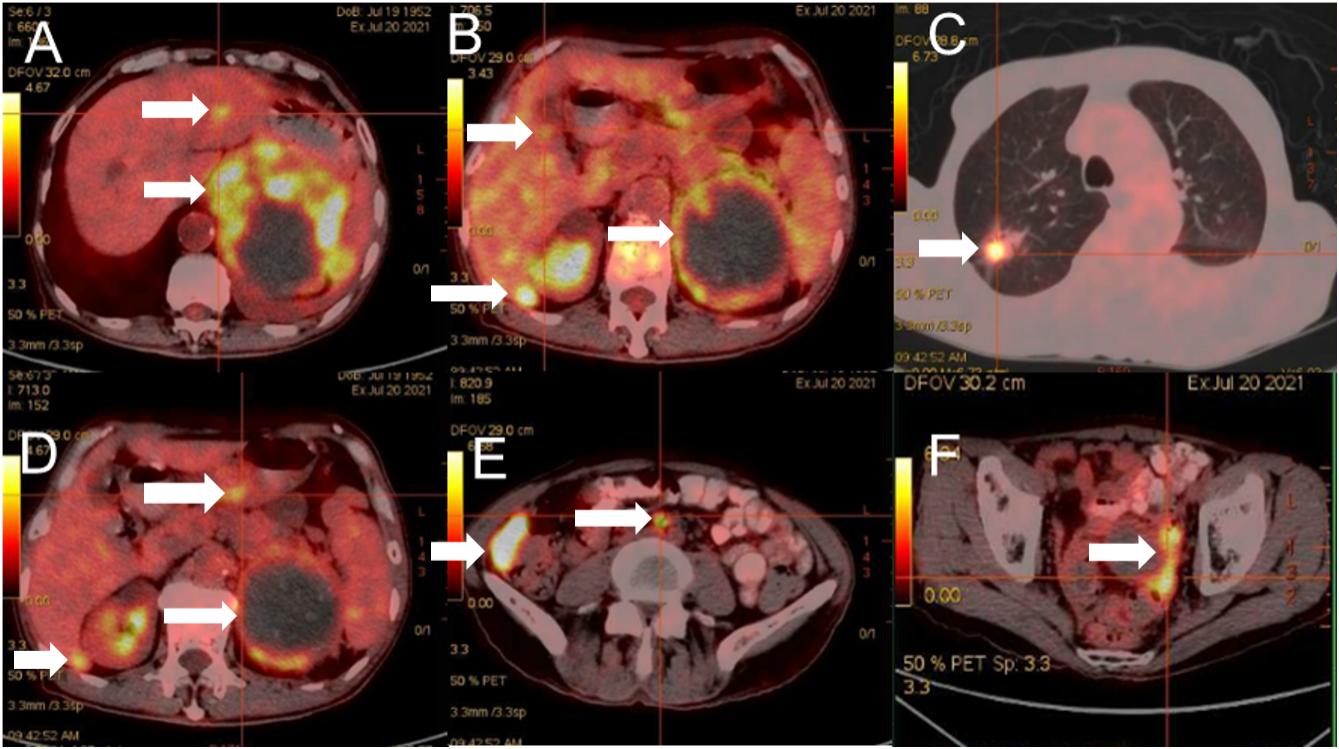
Positron emission tomography-CT showed a large, multi-loculated, hypermetabolic, cystic mass in the left adrenal region **(A-F)**.

**Figure 2:**
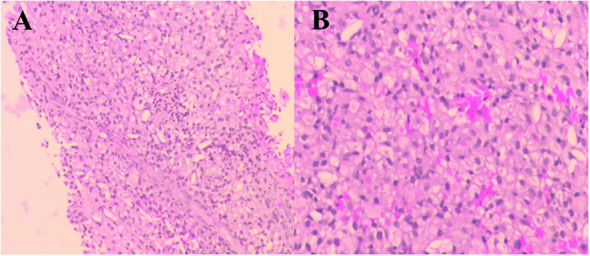
Pathological findings of the hypermetabolic mass (pelvic tissue). Hematoxylin and eosin (H&E) staining. Original magnification: **(A)**×40. **(B)** ×100.

**Figure 3:**
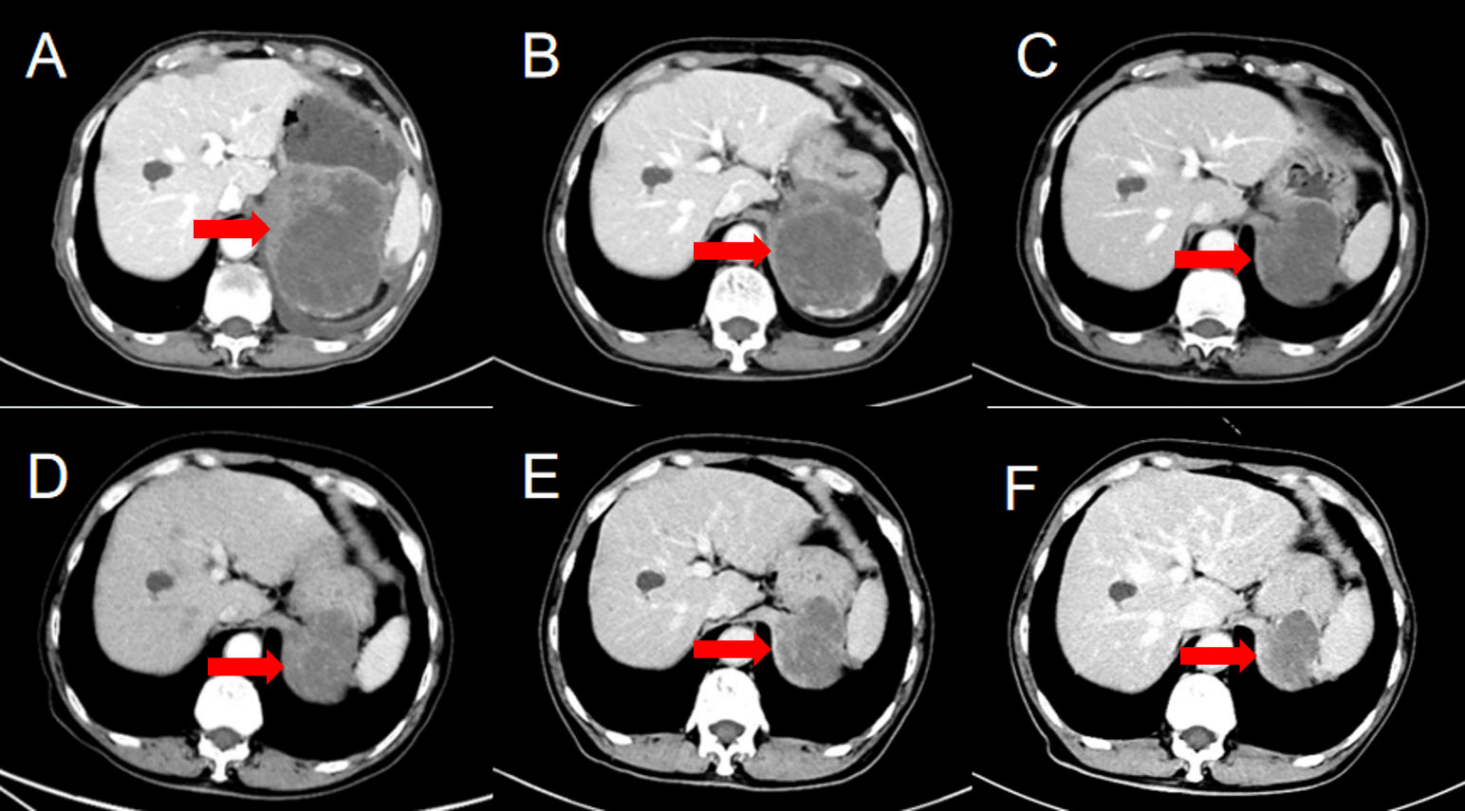
Computed tomography images before and after treatment. Review after immunotherapy showed significant reduction in the sizes of the left adrenal region. The time of CT was **(A)** 2021-07-30, **(B)** 2021-09-13, **(C)** 2021-11-30, **(D)** 2022-02-20, **(E)** 2022-05-12, **(F)** 2022-08-09.

A 69-year-old man was admitted to our hospital because of left-sided chest and abdominal pain for more than 2 months. His local hospital arranged computed tomography (CT) of the chest and abdomen. It was shown a huge mass in the left adrenal area, which was considered a possible malignant tumor, and multiple nodules in both lungs suggestive of metastatic cancer. His symptoms persisted, prompting referral to our hospital for further evaluation. The patient’s medical history included resection of a right retro-auricular meibomian cyst and left retro-auricular cystectomy. Our patient had no history of hypertension, diabetes and coronary heart disease, with no psychosocial abnormalities observed. The patient’s younger brother had a history of oral cancer. Physical examination: superficial lymph nodes were not palpably enlargement. Cardiopulmonary and abdominal examination did not show any significant abnormalities. Coagulation tests, urinalysis, stool analysis, urea, creatinine, uric acid, and an electrocardiogram were all within normal limits. Routine blood tests showed the white blood cell (WBC) was 3.35×10^9^/L, liver chemistry showed the alanine transaminase (ALT) was 67.8 U/L, and the aspartate aminotransferase (AST) was 126.9 U/L. CT of the chest and abdomen showed a mass in the left adrenal region, which was considered to be a malignant lesion. The mass was adjacent to stomach, kidney, and spleen, resulting in a high risk of puncture. Positron emission tomography-CT (PET-CT) was subsequently performed, which also showed a large, multi-loculated, hypermetabolic, cystic mass in the left adrenal region, and additionally showed hypermetabolic masses in the lung, peritoneum, liver, gallbladder, and multiple lymph nodes, suggesting distant metastasis. (**Figure 1**). Pathological examination of the hypermetabolic mass in pelvic tissue biopsy sample suggested epithelioid hemangioendothelioma (**Figure 2**). Immunohistochemical staining results were as follows: cytokeratin pan (−), Vimentin (+), PAX-8 (−), HMB45 (−), Syn (−), CgA (−), Ki-67 (approximately 30% +), S100 (−), CR (−), inhibin-α (−), CD31 (+), CD34 (−), ERG (+), TFE-3 (weak +), desmin (−), SMA (CKpan), and EMA (CKpan). The final diagnosis was a left suprarenal area EHE (Stage IV). Then, the patient was treated with 16 cycles of toripalimab (240 mg, intravenously (IV) days 1 once every 3 weeks) (**Table 1**). Periodic CT examinations showed significant reductions in the sizes of the metastases and primary lesion. Stable disease (SD) was achieved. Regular follow-up showed ongoing SD, and the disease had been stable for more than 13 months after initiation of treatment (**Figure 3**). During the treatment, no grade≥3 adverse events occured. Routine blood tests and liver chemistry showed the patient developed grade 1 anemia and grade 1-2 abnormal liver function. The patient is still alive now.

**Table 1:** The duration of medication administration.

Time	Drug
2021-07-07	toripalimab 240 mg
2021-07-30	toripalimab 240 mg
2021-08-24	toripalimab 240 mg
2021-09-14	toripalimab 240 mg
2021-10-09	toripalimab 240 mg
2021-11-08	toripalimab 240 mg
2021-12-01	toripalimab 240 mg
2021-12-28	toripalimab 240 mg
2022-01-24	toripalimab 240 mg
2022-02-21	toripalimab 240 mg
2022-03-18	toripalimab 240 mg
2022-04-15	toripalimab 240 mg
2022-05-13	toripalimab 240 mg
2022-06-14	toripalimab 240 mg
2022-07-12	toripalimab 240 mg
2022-08-10	toripalimab 240 mg

## Discussion

Epithelioid hemangioendotheliomas are rare, low-grade, malignant tumors that originate from vascular endothelial or pre-endothelial cells. The causes of this disease are unclear. In 2013, the WHO published guidelines for typing of sarcomas and proposed two types of EHE, one with a common classical gene t (1, 3) (p36.3; q25) translocation forming a *WWTR1-CAMTA1* fusion gene and the other with formation of an uncommon *YAP1-TFE3* fusion gene (1, 6–9). Previous studies have found that Ki-67 ≥10% is a predictor of poor prognosis (10). Simone et al. recommended that tumor size>3 cm appear to predict unfavorable outcomes (11). Our patient’s Ki-67 was approximately 30%+ and with a tumor size>3 cm, thus he likely had a poor prognosis. All tumors developed in association with immune deficiency. Accordingly, remodeling of antitumor immunity has been extensively studied and several therapeutic strategies for reshaping immune activity have been developed (12). Immune checkpoint modulation is the most important of these (13, 14). According to the European Society for Medical Oncology (ESMO) guideline of 2021, soft tissue sarcoma (STS) treatment options can be considered for highly invasive EHE: chemotherapy, targeted therapy, immunotherapy, etc. Toripalimab is one of the PD-1 (also known as PDCD1 or CD279) class of immune checkpoint inhibitors, which inhibit immune checkpoint receptors expressed on the surfaces of CD4+/CD8+ T lymphocytes and B lymphocytes (15). When PD-1 binds to programmed death ligand 1 (PD-L1)/programmed death ligand 2 (PD-L2), which is expressed on the surfaces of tumor cells, it inhibits T cell activation and proliferation and thus mediates negative immune regulation (16). Toripalimab binds with high affinity to PD-1 and selectively blocks the interaction between PD-1 receptor and ligand PD-L1, reactivating T cells and restoring their ability to kill tumor cells.

Because few EHEs have been examined pathologically and there are still too little clinical experiences with immunotherapy worldwide. There is no consensus on the optimal treatment of EHEs. A case report of epithelioid hemangioendothelioma occurs in both main bronchus and lung showed that the patient benefited from pembrolizumab and sirolimus, the potential target of immunotherapy might be POLE mutations (17). In our case, there was a giant tumor in the left adrenal region with multiple metastasis, which were unresectable. Considering that EHE was not sensitive to chemotherapy and radiotherapy, the immunotherapy agent toripalimab was used and good results achieved. A CT of the thoraco-abdominopelvic region performed after three cycles of immunotherapy showed that the left retroperitoneal mass had reduced in size from 100 mm×85 mm×115 mm to 77 mm×85 mm×107 mm. Another CT of the thoraco-abdominopelvic region after six cycles of immunotherapy showed that the mass had reduced further in size to 80 mm×75 mm×96 mm. After 15 cycles of immunotherapy, the size of the mass was 84 mm×65 mm×92 mm, and the overall assessment was SD. But another patient with pulmonary epithelioid hemangioendothelioma didn’t response to sintilimab and chemotherapy. The poor response to the treatment may be because the patient harbored a germline PALB2 mutation and WWTR1-CAMTA1 gene fusion (18). Thus, gene sequencing should be performed to help the decision of treatment. However, our patient’s financial situation was poor, gene sequencing was not performed. For further investigation, gene sequencing may be performed.

During the treatment of toripalimab, the adverse event was tolerable and well controlled. The patient’s Electrocardiogram (ECG) showed the heart rate dropped gradually, sinus bradycardia was discovered. After 12 cycles of treatment, dynamic ECG was performed to evaluated the cardiac side effect and showed multiple variations: sinus rhythm; supraventricular asystole, occasionally with poor transmission and short paroxysmal supraventricular tachycardia; occasional ventricular asystole; and no significant abnormal dynamic changes in ST-T. The slowest heart rate was 47 beats/min and the fastest was 129 beats/min, with an average of 59 beats/min. No other adverse effects.

## Conclusion

In conclusion, we presented a case of advanced left suprarenal area EHE patient. The patient was treated with toripalimab and achieved a SD, with a long-term PFS of more than 13 months. To our knowledge, this is the first report of retroperitoneal EHE treated with PD-1 inhibitor. The PD-1 inhibitor toripalimab may be a promising agent for the treatment of advanced EHE. However, further prospective and controlled trials with large number of patients are needed to assess the safety and efficacy of toripalimab in EHE, as well as to find potential efficacy predictive targets.

## Data availability statement

The original contributions presented in the study are included in the article/supplementary material. Further inquiries can be directed to the corresponding author.

## Ethics statement

Written informed consent was obtained from the individual for the publication of any potentially identifiable images or data included in this article.

## Author contributions

YB drafted the paper. HZ, LP, and XN revised the paper. Pathological images are provided by BL, ML, LH, and FW did literature search. All authors contributed to the article and approved the submitted version.
